# Clinical diagnosis and treatment of subungual exostosis in children

**DOI:** 10.3389/fped.2022.1075089

**Published:** 2022-12-08

**Authors:** Hao Li, Haichong Li, Xinyu Qi, Dong Guo, Jun Cao, Yunsong Bai, Ziming Yao, Xuejun Zhang

**Affiliations:** Department of Orthopedics, Beijing Children’s Hospital, Capital Medical University, National Center for Children’s Health, Beijing, China

**Keywords:** subungual exostosis, children, nail bed, surgical treatment, treatment effect

## Abstract

**Objective:**

To analyze and summarize the clinical characteristics and treatment effects for subungual exostosis in children.

**Methods:**

Clinical data for children with subungual exostosis treated in our department from January 2008 to September 2022 were evaluated.

**Results:**

Forty children with subungual exostosis were evaluated, comprising 31 boys (77.5%) and 9 girls (22.5%) with a median age of 9 years (4–17 years). The median disease course was 6 months (1–48 months). Seven patients (17.5%) had definite trauma history and 5 (12.5%) had infection. The toe or finger nail appearance was abnormal in 36 patients and normal in 4 patients. Twenty-seven patients (67.5%) had pain when wearing shoes and walking, and 25 (62.5%) had toenail tenderness. The lesions were located in the distal phalanxes of the toes in 37 patients (92.5%), with 14 patients affected on the left side and 23 on the right side. Twenty-two patients had lesions in the great toe, 6 in the second toe, 6 in the third toe, and 3 in the fourth toe. The lesions in the other 3 patients (7.5%) were located in the distal phalanxes of the fingers, with 2 patients affected in the second finger and 1 in the third finger. Regarding the relationship between lesion location and nail bed, 4 patients were type I, 21 were type II, and 15 were type III. All 40 patients received surgical treatment, with nail removal in 15. The median maximum lesion diameter was 1.0 cm (0.8–2 cm), median operation time was 25 min (20–45 min), median blood loss was 1 ml (1–2 ml), and median postoperative hospital stay was 2 days (1–4 days). All cases were histopathologically confirmed as subungual exostosis. The median follow-up time was 24 months (3–60 months), with normal appearance of the toe or finger nail. There were no complications in 38 patients (95.0%). Two patients (5.0%) relapsed at 3 months postoperatively and underwent a secondary operation, with no subsequent recurrence during 12 months of follow-up.

**Conclusion:**

Subungual exostosis in children is a rare benign disease that often occurs in the toes. Selection of the appropriate incision and nail bed treatment based on the relationship between lesion location and nail bed is helpful for improving the treatment effect.

## Introduction

Subungual exostosis is a relatively rare osteochondroma that affects the distal phalangeal bones of the toes or fingers (1). The main symptoms are pain and nail bed or nail deformity. In severe cases, subungual exostosis causes pain when wearing shoes and walking, and affects study and daily life. Although the etiology remains unclear, it is thought to be related to minimally invasive injury and infection (2). The disease was first described by Dupuytren (3) who observed subungual exostosis of the toe. This benign lesion was reported to be a part of multiple hereditary exochondroma (1, 4), although its pathology differs from that of osteochondroma. Because of the poor effects of conservative treatment, surgery is the ultimate and effective treatment. The present study aimed to retrospectively summarize and analyze the clinical data of children with subungual exostosis treated in our department.

## Materials and methods

### Subjects

From January 2008 to September 2022, 40 children with subungual exostosis were treated surgically and confirmed pathologically in our department. Their clinical data, including sex, age, trauma history, infection history, nail appearance, lesion location, relationship between lesion location and nail bed, and surgical treatment process, were recorded. The study was approved by the Ethics Committee of Beijing Children's Hospital, Capital Medical University, National Center for Children's Health, Beijing, China.

### Clinical classification

Using anteroposterior and lateral x-ray films of the toes or fingers, and the relationship between lesion location and nail bed was evaluated. Based on this relationship, the lesions were divided into three types (Table 1): Type I, lesion located at the distal phalangeal bone margin, without invasion of nail bed, with no obvious protrusion at edge of nail, and without slight protrusion or deformation of nail. Type II, lesion located at the distal phalangeal bone margin, with slight invasion of nail bed, obvious protrusion at edge of nail, and protrusion and deformation of nail. Type III, lesion located at the dorsal side of the phalange bone has a wide base, obvious invasion of nail bed, and deformation of nail (nail obviously uplifted and deformed).

**Table 1:** Types of subungual exostosis.

	Location of lesion	Nail bed invasion	Selection of operation methods
Type I	Distal phalangeal bone margin	No invasion of nail bed	The skin incision on the edge of nail bed was not removed
Type II	Distal phalangeal bone margin	Slight invasion of nail bed edge	Without pulling out the nail, the edge of nail bed was directly removed
Type III	The dorsal side of the phalange bone has a wide base	Obvious invasion of nail bed	Remove the nail, remove the lesion and repair the nail bed

### Selection of operation methods

All 40 patients underwent surgical treatment with good outcomes: 4 patients with type I lesions underwent surgery through a skin incision at the edge of the nail bed without pulling out the nail; 21 patients with type II lesions underwent surgery without pulling out the nail and had the lesion directly removed at the edge of the nail bed after trimming the nail; and 15 patients with type III lesions underwent surgery by pulling out the deck, removing the lesion, and repairing the nail bed. The operation methods, maximum lesion diameter, operation time, blood loss, and postoperative hospital stay were recorded. The final diagnosis of all patients were confirmed by histopathological examination.

### Follow-up

Patients were followed up at 1, 3, 6, and 12 months postoperatively and once a year thereafter. The nail appearances and x-ray films of the toes or fingers were evaluated at each follow-up.

### Statistical analysis

SPSS 24.0 for Windows (IBM Corp., Armonk, NY, USA) was used for statistical analyses. Continuous variables were presented as median (minimum–maximum) and categorical variables were presented as number of cases (percentage).

## Results

### General information and medical history

From January 2008 to September 2022, a total of 40 children with subungual exostosis were treated surgically and confirmed by postoperative pathology, comprising 31 boys (77.5%) and 9 girls (22.5%). The median age of the patients was 9 years (4–17 years), and the median disease course was 6 months (1–48 months). Seven patients (17.5%) had definite trauma history (5 with kick injury, 2 with collision injury in bath), 23 (57.5%) had possible trauma history (low grade recurrent injury as on wearing tight ill fitted shoes), and 10 (25.0%) had no trauma history. Five patients (12.5%) had infection, 30 (75.0%) were confirmed to have no infection, and 5 (12.5%) had possible infection.

The toe or finger nail appearance was abnormal in 36 patients and normal in 4 patients. Twenty-seven patients (67.5%) had pain when wearing shoes and walking that also affected study and daily life, and 25 (62.5%) had toenail tenderness. Thirty-nine patients had single subungual exostosis only, and 1 had subungual exostosis complicated with multiple osteochondroma of the bilateral scapulae and ribs. The general data for the patients are shown in Table 2.

**Table 2:** General data for the patients.

	Gender		History of trauma			History of infection			Nail deformity		Pain		Tenderness		Nail removal		Nail bed treatment		Relapse		Total
	male	female	Yes	Possible	No	Yes	Possible	No	Yes	No	Yes	No	Yes	No	Yes	No	Repair and suture	Protection of nail bed	Yes	No	
Number of patients	31	9	7	23	10	5	5	30	36	4	27	13	25	15	15	25	15	25	2	38	40
Percentage (%)	77.5	22.5	17.5	57.5	25.0	12.5	12.5	75.0	90.0	10.0	67.5	32.5	62.5	37.5	62.5	37.5	62.5	37.5	5.0	95.0	100

### Locations of subungual exostosis

The lesions were located in the distal phalanxes of the toes in 37 patients (92.5%), with 14 patients affected on the left side and 23 on the right side. Twenty-two patients (55.0%) had lesions located in the great toe, 6 (15.0%) in the second toe, 6 (15.0%) in the third toe, and 3 (7.5%) in the fourth toe. The lesions in the other 3 patients (7.5%) were located in the distal phalanxes of the fingers, with 2 patients affected in the second finger and 1 in the third finger. Regarding the relationship between lesion location and nail bed, 4 patients (10.0%) were type I, 21 (52.5%) were type II, and 15 (37.5%) were type III. Radiographically, the dorsal bone of the distal toe (finger) was overgrown and composed of trabeculae. Twenty-five patients (62.5%) had lesions located in distal phalangeal bone margin with narrow bases, and 15 patients (37.5%) had lesions located in dorsal side of the phalange bone with wide bases.

### Surgical treatment

**Figure 1:**
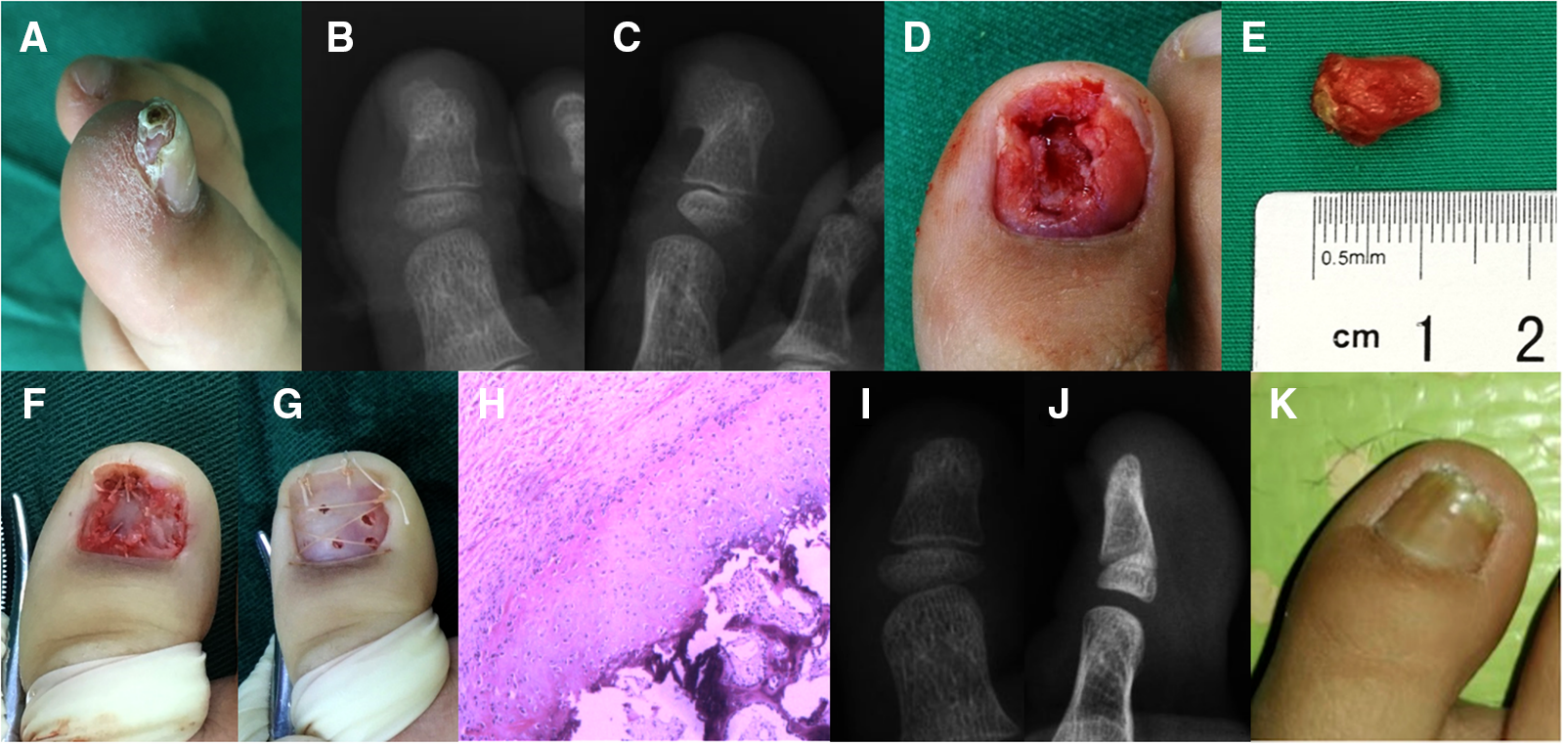
Subungual exostosis of the great toe in a 7-year-old boy. (**A**) Physical examination revealed a hard protuberant lesion measuring 1.2 × 0.8 cm under the right great toe. (**B,C**) Radiographically, there was a calcified projection on the dorsomedial side of the distal phalanx, which was continuous with the normal underlying bone. (**D,E**) The nail bed was invaded by the lesion and the exostectomy was performed. (**F**) The damaged nail bed was repaired by cutting a thin layer of tissue from the normal nail bed. (**G**) Replantation of the toenail was adopted. (**H**) Histopathological analysis (hematoxylin-eosin staining, ×40 magnification) revealed normal trabecular bone and fibrocartilage were overgrown, these findings were consistent with the diagnosis of subungual exostosis. (**I–K**) No recurrence was found on posteroanterior and lateral radiographs, and the appearance of the toenail remained good during 12 months of follow-up. The patient's parents provided written informed consent to publish the details of the patient's case.

**Figure 2:**
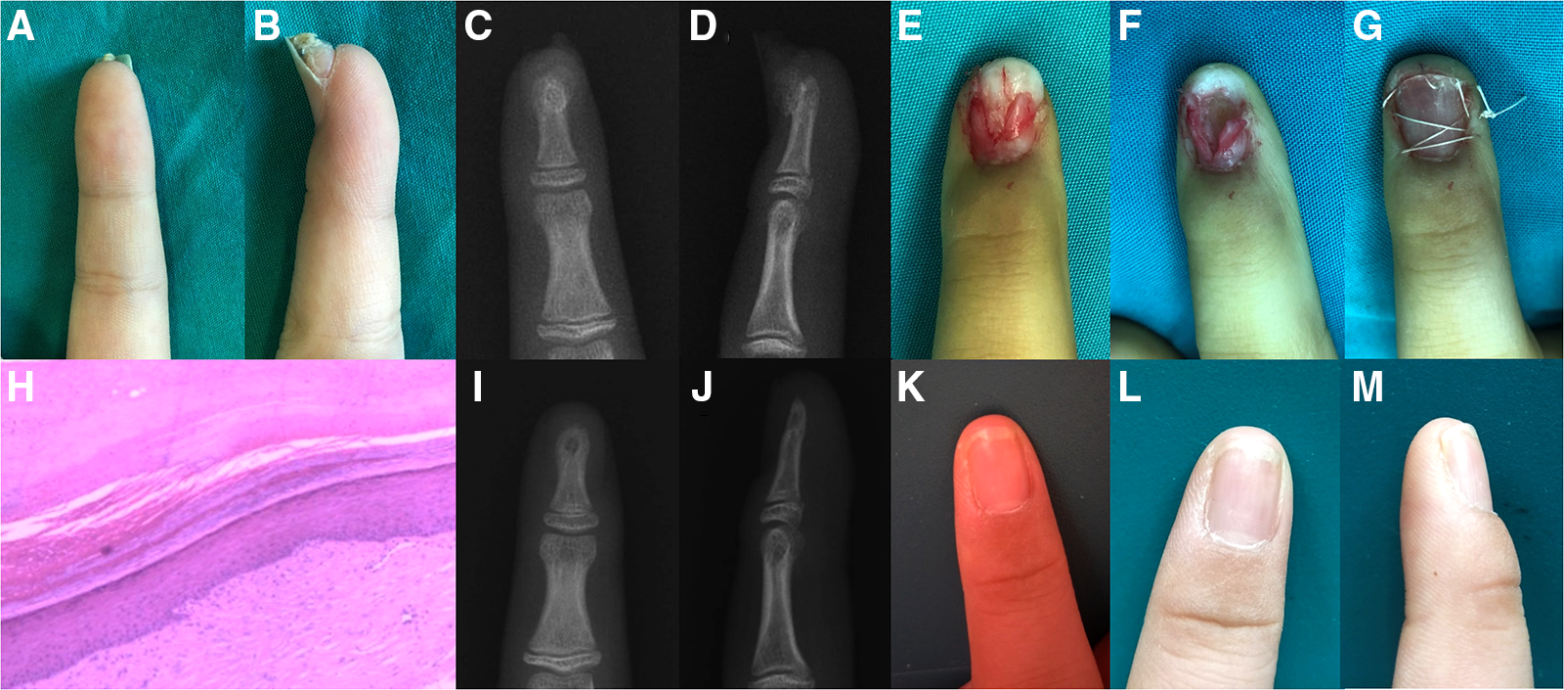
Subungual exostosis of the finger in an 8-year-old girl. (**A,B**) Physical examination revealed a firm, protuberant lesion of 0.8 ×  0.5 cm under the right index finger. (**C,D**) Posteroanterior and lateral radiographs of the lesion. (**E**) The lesion invaded the nail bed. (**F**) The lesions were completely removed with sufficient margin. (**G**) Replantation of the fingernail was adopted. (**H**) Histopathological analysis (hematoxylin-eosin staining, ×40 magnification) revealed normal trabecular bone and fibrocartilage were overgrown, these findings were consistent with the diagnosis of subungual exostosis. (**I–M**) No recurrence was found on posteroanterior and lateral radiographs, and the appearance of the fingernail remained good during 15 months of follow-up. The patient's parents provided written informed consent to publish the details of the patient's case.

All 40 patients received surgical treatment. Fifteen patients (37.5%) had nail removal and 25 patients (62.5%) had no nail removal. Fifteen patients (37.5%) had repair involving suture of the nail bed. If the nail bed could not be sutured directly, a thin layer of normal nail bed tissue was cut as a patch to repair and suture the nail bed. The median maximum lesion diameter was 1.0 cm (0.8–2 cm), median operation time was 25 min (20–45 min), median blood loss was 1 ml (1–2 ml), and median postoperative hospital stay was 2 days (1–4 days). The maturation of cartilaginous and bone trabeculae with spindle cell proliferation can be seen histopathologically, and all cases were confirmed as subungual exostosis. The clinical data and treatment of with subungual exostosis are presented in Figures 1, 2.

### Follow-up

The median follow-up period was 24 months (3–60 months). At the last follow-up, the daily activities and movements of the patients were unaffected, the toe or finger nail appearance was normal, and the children and their parents were satisfied. Thirty-eight patients (95.0%) had no complications such as toe or finger necrosis, toe nail deformation, recurrence, and functional abnormalities. The other 2 patients (5.0%) had recurrence at 3 months after the primary resection, and underwent a secondary resection of the lesion by nail bed separation. No further recurrence was observed in these patients during 12 months of follow-up.

## Discussion

Subungual exostosis is a benign slow-growing exochondroma of the toe (finger), which is rare in the general population and children. Dupuytren (3) first described the disease of subungual exostosis of the distal phalanx in 1847, pointing out that it is a benign proliferative osteochondroma firmly attached to the distal toe (finger) bone, and subsequent reports showed benign bone hyperplasia of the distal toe (finger) with unknown etiology (5, 6). In children and young people, subungual exostosis usually presents as an isolated small solid lesion, located deep in the free edge of the toenail (7). Although chronic stimulation is considered the cause of fibrocartilage metaplasia, the pathogenesis of subungual exostosis remains unclear. Most clinicians believe that subungual exostosis is a reactive metaplasia caused by microtrauma (8). However, there is no conclusive evidence to support this single pathogenesis (9). Many possible factors are related to the formation of subungual exostosis, including chronic infection, trauma, genetic abnormalities, tumors, and cartilage cyst activation (10). Some studies have suggested that trauma is the main factor in the formation of subungual exostosis, followed by acute and chronic inflammation leading to chondrometaplasia (11). In the present study, 7 patients had definite trauma history, 23 had possible trauma history, and 10 had no trauma history, while 5 had infection, 30 had no infection, and 5 had possible infection.

The prevalence of subungual exostosis is unclear and may be underestimated, because its clinical manifestations are unclear and inconsistent, often leading to delayed diagnosis (7). Most related injuries occurred in the toe, while injuries to other toes were less common (12) and only a few injuries occurred in the fingers (13). The most common injury site was the middle or medial part of the toe (6).

Subungual exostosis usually presents as a mass protruding from under the nail bed. In some patients, pain, ulcer, nail bed infection, and secondary changes to the surrounding tissues can also occur, leading to adverse effects on their health-related quality of life (14). Subungual exostosis usually presents as a hard fixed nodule under the nail at the distal end of the toe (finger) bone, with hyperkeratosis on the surface. As the subungual exostosis grows, the toenail is lifted and becomes separated from the nail bed below. Pain and infection are the most common complications, and represent chronic and progressive processes (15). In the present study, there were 36 patients with toenail deformity and 25 patients with toenail tenderness, consistent with the previous report (15).

It is difficult to diagnose subungual exostosis by clinical manifestations alone. Misdiagnosis or delayed diagnosis is common. In one study, the incidence of delayed diagnosis was about 10%, and more than 10% of patients had toenail dystrophy (7). The main complaint of patients with subungual exostosis was nail pain, and the diagnosis was confirmed by imaging. x-ray examination is a noninvasive diagnostic method that forms part of the preliminary examination of any suspected subungual tumor (6). x-rays showed that the dorsal bone of the distal toe (finger) was overgrown and composed of trabeculae. Magnetic resonance imaging can detect different signal structures in tumors, which is helpful for the diagnosis of subungual exostosis (16). Although x-ray examination and histopathological examination are necessary for the diagnosis, dermoscopy is a useful auxiliary tool for the diagnosis of this benign toenail disease (17). Dermoscopy can distinguish subungual exostosis from other toenail diseases, and can be used to guide the treatment of patients (18). The differential diagnosis of subungual exostosis includes squamous cell carcinoma of the nail bed, verruca vulgaris, glomus tumor, subungual fibroma/fibrokeratoma, subungual epidermal inclusion body cyst, suppurative granuloma, pigmented malignant melanoma, melanoma, osteosarcoma, and endochondroma (11). Subungual exostosis should be considered in the differential diagnosis of any painful subungual tumor, especially when there is a yellow area on the deck above the subungual mass (19). Endochondroma has a similar clinical appearance, but can be radiographically transmitted and cause expansion of the phalanx itself (20). In addition, there are some differences in the ossification process and histopathology between subungual exostosis and subungual osteochondroma. However, when treated appropriately, the lesions have good functional and cosmetic results, as well as a very low recurrence rate (7).

When the disease is serious, it will affect children's shoe wearing, walking, and daily life. Conservative treatment is ineffective, and surgical resection is the first choice. The main purpose of the surgical treatment is to remove the exostosis to the boundary of normal bone tissue to prevent recurrence, and to avoid toe nail deformity by protecting the nail bed and nail matrix. The success rate of surgical resection was more than 90% (21). It was reported that 53% of recurrences were related to inadequate tumor resection (22). Resection of the entire lesion, including the fibrocartilage cap, is essential to avoid local recurrence (23). Another report described recurrence of subungual exostosis after surgery, followed by spontaneous subsidence (24). Complete resection of the lesion and fine separation from the nail bed structure can significantly reduce the recurrence rate and complications (25). During surgery for subungual exostosis, it is very important to remove the mass without damaging the nail bed. However, if the nail bed is damaged during the operation, it should be repaired after removal of the mass. Two of the 40 patients in the present study experienced recurrence, giving a surgical success rate of 95.0% and a recurrence rate of 5.0%. The reason for the recurrence may be related to incomplete resection during the first operation.

Regarding selection of the surgical incision and protection of the nail bed, there are many reports on surgical methods (26, 27). Suga and Mukouda provided treatment suggestions according to whether the nail bed was damaged and the size of the lesion (9). Specifically, if the lesion involved the nail bed and was >5 mm, they recommended to split the nail bed and cover it with an artificial skin. However, all of their patients had ingrown nail deformity after the surgery. Morato (28) divided lesions into four types based on the patient's symptoms, toenail involvement, and lesion location: type 1, mild deformity, the deck and nail bed are basically normal, and it is recommended to make a fish-mouth incision at the end of the toe; type 2, the lesion is located at the far end of the deck, with distal medullary hyperplasia and ingrown nail deformity, and it is recommended to make an oval double incision; type 3, the lesion has severe curved nail plate (pincer nail), dystrophic distal nail plate, associated onychocryptosis, and pain associated with nail destruction; type 4, the lesion is associated with onychocryptosis, pain in the nail border or proximal nail fold, and a bony protrusion located below or inside and outside the toenail. According to the classification, different surgical treatments are performed. The surgical methods selected depend on the size, shape, and location of the lesion. However, we found that some lesions could not be classified into these four types. Therefore, according to our case data and experience, combined with the location of the subungual exostosis and the impact on the nail bed, the nail bed treatment methods were classified to guide the choice of surgical incision and nail bed treatment methods. Regarding the surgical methods used in the present study, 4 patients with type I lesions underwent treatment through a skin incision at the edge of the nail bed while retaining the nail, 21 patients with type II lesions underwent direct removal of the edge of the nail bed after trimming the nail, and 15 patients with type III lesions had removal of the deck, followed by resection of the lesion and subsequent repair of the nail bed. The data for these 38 patients proved that the lesion could be fully exposed and completely removed, and the nail bed could be successfully healed and shaped. Meanwhile, 2 patients with recurrence required a secondary operation.

## Conclusions

Subungual exostosis is a rare benign disease that is usually difficult to diagnose in the early stage, leading to delays in diagnosis and treatment. x-ray examination can be used for the differential diagnosis of subungual exostosis. Surgical resection is the main treatment. Complete resection of the lesion and careful separation from the nail bed structure can significantly reduce the risk of recurrence. Based on the clinical types, different surgical methods should be selected to remove the lesions and deal with the nail bed. Damage to the nail bed and nail matrix should be minimized to avoid deformity of the nail.

## Data Availability

The raw data supporting the conclusions of this article will be made available by the authors, without undue reservation.
